# A molecular targeting against nuclear factor-*κ*B, as a chemotherapeutic approach for human malignant mesothelioma

**DOI:** 10.1002/cam4.202

**Published:** 2014-02-10

**Authors:** Sho Nishikawa, Akane Tanaka, Akira Matsuda, Kumiko Oida, Hyosun Jang, Kyungsook Jung, Yosuke Amagai, Ginae Ahn, Noriko Okamoto, Saori Ishizaka, Hiroshi Matsuda

**Affiliations:** 1 Cooperative Major in Advanced Health Science, Graduate School of Bio-Applications and System Engineering, Tokyo University of Agriculture and TechnologyFuchu, Tokyo, Japan; 2 Laboratory of Comparative Animal Medicine, Tokyo University of Agriculture and TechnologyFuchu, Tokyo, Japan; 3 Laboratory of Veterinary Molecular Pathology and Therapeutics, Division of Animal Life Science, Institute of Agriculture, Tokyo University of Agriculture and TechnologyFuchu, Tokyo, Japan

**Keywords:** Animal model, cell cycle, mesothelioma, NF-*κ*B

## Abstract

Chronic inflammation due to the absorption of asbestos is an important cause of mesothelioma. Although the increased prevalence of mesothelioma is a serious problem, the development of effective chemotherapeutic agents remains incomplete. As the nuclear factor-*κ*B (NF-*κ*B) pathway contributes to malignant transformation of various types of cells, we explored NF-*κ*B activity in three different pathological types of malignant mesothelioma cells, and evaluated the therapeutic potential of a recently reported NF-*κ*B inhibitor, IMD-0354. NF-*κ*B was constantly activated in MSTO-211H, NCI-H28, and NCI-H2052 cells, and the proliferation of these cell lines was inhibited by IMD-0354. D-type cyclins were effectively suppressed in mixed tissue type MSTO-211H, leading to cell cycle arrest at sub G_1_/G_1_ phase. IMD-0354 reduced cyclin D3 in both epithelial tissue type NCI-H28 and sarcomatoid tissue type NCI-H2052. In a sphere formation assay, IMD-0354 effectively decreased the number and diameter of MSTO-211H spheres. Preincubation of MSTO-211H cells with IMD-0354 delayed tumor formation in transplanted immunodeficient mice. Furthermore, administration of IMD-0354 markedly rescued the survival rate of mice that received intrathoracic injections of MSTO-211H cells. These results indicate that a targeted drug against NF-*κ*B might have therapeutic efficacy in the treatment of human malignant mesothelioma.

## Introduction

Malignant mesothelioma is one of the most severe life-threatening diseases in the pleura, and it has strong causal relationships with chronic inflammation against foreign substances, primarily asbestos. It is convincing that almost all mesotheliomas are initiated by chronic inflammation and fibril formation following exposure to asbestos 1,2. In addition, there is an incubation period of decades from asbestos exposure to the onset of mesothelioma 1,2, and it is assumed that the number of patients with mesothelioma will increase within the next few decades because of the worldwide peak use of asbestos in the 20th century 3–6. Patients with mesothelioma often display increasing pleural effusion containing clusters of tumor cells, and these cells are thought to metastasize and form space-occupying lesions, resulting in breathing difficulty. As mesothelioma remains a lethal and therapy-resistant disease 3–6, the establishment of an effective therapeutic strategy is essential. Although much of the precise mechanism of the pathogenesis of mesothelioma remains unknown, the involvement of chronic inflammation in the pathogenesis has been proposed. After mesothelial cells and macrophages phagocytose asbestos, these cells produce pro-inflammatory cytokines such as interleukin-8 (IL-8), IL-1*α*, IL-1, IL-6, and tumor necrosis factor-*α* (TNF-*α*) 3,7,8. Although the uptake of asbestos often induces cytotoxicity and results in the apoptosis of affected cells, TNF-*α* produced by macrophages may rescue mesothelial cells from cell death through a nuclear factor-*κ*B (NF-*κ*B)-dependent pathway 8. In addition, asbestos is thought to stay long in the respiratory system and sustain the chronic inflammation in microenvironmental tissues including adipose tissue, where adipocytes secrete some adipocytokines promoting pro-inflammatory reaction 9. These global inflammatory environments have been considered to induce the transformation of mesothelial cells and progression of mesothelioma.

NF-*κ*B is a dimeric transcription factor of the Rel family. Generally, NF-*κ*B exists in an inactive form in the cytoplasm by binding to an intrinsic inhibitor, I*κ*B family proteins 10. Once I*κ*B is phosphorylated, NF-*κ*B translocates from the cytoplasm to the nucleus, after which, NF-*κ*B binds to specific DNA sequences of target genes and induces cell proliferation and various cell functions 10–13. In previous studies, constitutive activation of NF-*κ*B was observed in some tumor cells 14–17, suggesting that NF-*κ*B is a potential therapeutic target 18,19. We previously reported the substantial involvement of constitutive activation of NF-*κ*B in the neoplastic proliferation of mast cells, breast cancer cells, and canine leukemia cells 20–22. In breast cancer cells in particular, we demonstrated that NF-*κ*B activation was related to the expression of cyclins D1, D2, and D3, which accelerated the effects of cyclin-dependent kinase (CDK) on cell division concurrent with the phosphorylation of retinoblastoma protein. In addition, NF-*κ*B inhibition with a specific inhibitor of I*κ*B kinase *β* (IKK*β*), IMD-0354, suppressed cell cycle progression in breast cancer cells, leading to apoptotic cell death 20. Study findings revealed the antitumor potential of an NF-*κ*B inhibitor in cancers in which NF-*κ*B substantially contributes to pathogenesis. Exposure to asbestos induces chronic inflammation that is associated with activation of NF-*κ*B signaling and subsequent production of TNF-*α* and IL-1 23. Crucial roles of NF-*κ*B signaling in both malignant transformations have been identified 24. However, direct evidence regarding NF-*κ*B in triggering mesothelioma formation has been insufficient in clinical cases.

Given the involvement of NF-*κ*B in asbestos-related inflammation and the subsequent tumorigenic transformation of mesothelial cells, drugs that target NF-*κ*B may become promising candidates for the treatment of mesothelioma. Few reports revealed that NF-*κ*B was activated in mesothelioma cells and the use of proteasome inhibitors or antitumor ribonuclease reagents was effective in controlling tumorigenic proliferation and inducing an apoptotic reaction through the prevention of NF-*κ*B activity 21,22,25,26. However, these reagents may influence the degradation and synthesis of a wide range of functional proteins. Therefore, the development of effective drugs with fewer side effects is necessary. Although previous reports suggested serious roles of NF-*κ*B activation in mesothelioma, only fragmented information has been provided. Thus, more detailed investigation of NF-*κ*B activation relating to tumor progression might be necessary and significant for the establishment of therapy with NF-*κ*B regulation.

In this study, we detected sustained NF-*κ*B activation in three different pathological types of mesothelioma cells. IMD-0354 effectively prevented the activation of NF-*κ*B and the progression of mesothelioma cells. In addition, IMD-0354 successfully suppressed the expression of D-type cyclins, resulting in cell cycle arrest. NF-*κ*B inhibition by IMD-0354 also downregulated sphere formation in mesothelioma cells. Furthermore, NF-*κ*B inhibition effectively suppressed tumor expansion in two transplantation models of mesothelioma. This study revealed that NF-*κ*B may seriously contribute to tumor formation and progression in mesothelioma and that its inhibition will be an effective therapeutic strategy with variations in the cancer hierarchy.

## Materials and Methods

### Cell culture

Three different human malignant mesothelioma cell lines, MSTO-211H (mixed tissue type), NCI-H2052 (sarcomatoid tissue type), and NCI-H28 (epithelial tissue type), were purchased from American Type Culture Collection (Manassas, VA) and cultured in RPMI 1640 supplemented with 10% fetal bovine serum and 1% penicillin–streptomycin.

### Reagents

Rabbit anti-phospho-I*κ*B*α* antibody, rabbit anti-I*κ*B*α* antibody, and rabbit anti-*β*-actin antibody were obtained from Cell Signaling Technology (Beverly, MA). Rabbit anti-NF-*κ*B p65 antibody, anti-Bcl-2 antibody, and anti-Histon H1 antibody were purchased from Santa Cruz Biotechnology (Lake Placid, NY). Rabbit anti-cyclin D1 antibody, anti-cyclin D2 antibody, anti-cyclin D3 antibody, and anti-cyclin E antibody were purchased from PharMingen (San Diego, CA). Horseradish peroxidase-conjugated secondary antibodies were obtained from Jackson ImmunoResearch Laboratories, Inc. (West Grove, PA). IMD-0354, an IKK*β* inhibitor 20–22, was kindly provided by the Institute of Medicinal Molecular Design Inc. (Tokyo, Japan). Pemetrexed and cisplatin were purchased from LKT Laboratories, Inc. (St. Paul, MN).

### Western blot analysis

After incubation in a serum deprivation condition for 12 h, the medium was changed to serum-containing medium, and simultaneously, cells were exposed to various concentrations of IMD-0354. In total, 2 × 10^6^ cells were collected after 0, 3, 6, 9, and 12 h of treatment. Cells were washed with phosphate-buffered saline (PBS) and lysed in 100 *μ*L of CelLytic-M reagent supplemented with a protease inhibitor cocktail (Sigma Chemicals, St. Louis, MO). Nuclear and cytoplasmic extraction, Western blot analysis, and the calculation of relative intensities were performed as previously described 20.

### 3-[4,5-Dimethylthiazol-2-yl]-2,5-diphenyltetrazolium bromide (MTT) assay

Cells (1 × 10^5^ cells/mL) were applied to each well of 96-well culture plates and incubated in 100 *μ*L of serum-containing RPMI1640 medium in the presence or absence of various concentrations of IMD-0354, cisplatin (Cis, 0.01 *μ*g/mL), and pemetrexed (Pem, 0.1 *μ*g/mL) for 48 h. Beginning at 4 h before the end of culture, 10 *μ*L of 5 mg/mL MTT dissolved in PBS was added to each well. The reaction was stopped by the addition of 100 *μ*L of 10% sodium dodecyl sulfate in 0.01 mol/L HCl. Absorbance was measured at 577 nm using ImmunoMini NJ-2300 (Nalge Nunc International K.K., Tokyo, Japan). The inhibitory rate of proliferation was calculated by the following formula: Inhibitory rate of proliferation = 1 − (average optimal density [OD] in the treatment group/average OD value in the control group).

### Cell cycle analysis

After the incubation of cells (2 × 10^5^ cells/mL) in the presence or absence of IMD-0354 (1.25 *μ*mol/L) or pemetrexed (0.1 *μ*g/mL) for 24 h, cells were collected, washed twice with ice-cold PBS, and fixed in 70% ethanol for 30 min with periodic vortexing. Cells were collected and treated with 0.5 *μ*g/mL RNase A for 20 min at 37°C to avoid nonspecific propidium iodide (PI) binding. DNA was stained by incubating cells in 50 *μ*g/mL PI dissolved in 0.1% sodium citrate for 10 min on ice with periodic vortexing. To remove cell clustering, cells were passed through a nylon mesh. DNA contents were analyzed using a flow cytometer (Coulter, Hialeah, FL).

### Terminal deoxynucleotidyl transferase-mediated dUTP nick end-labeling assay (TUNEL assay)

Cells (1 × 10^5^ cell/mL) were applied to each well of 8-well Permanox slide (Lab-Tek® chamber slideTM System, Thermo Fisher scientific Inc., NY) and incubated in 200 *μ*L of serum-containing RPMI1640 medium in the presence or absence of IMD-0354 (1.25 *μ*mol/L) and pemetrexed (0.1 *μ*g/mL) for 24 h. After the incubation, the cells were fixed immediately in 4% formaldehyde at room temperature for 1 h. Then, TUNEL assay was performed with DeadEndTM Colormetric TUNEL System (Promega, Madison, WI) according to the manufacturer's instruction. The number of positive-labeled cells stained as dark brown was counted in five randomly selected fields under a microscope (200× magnification) and expressed as percentage of total cells counted (at least 500 cells).

### Sphere formation assay

Cells (3 × 10^5^ cells/mL) were seeded in 6-well plates and incubated in serum-free medium with or without various concentrations of IMD-0354 for 3 days. To avoid suppression of NF-*κ*B activity, nutrition including AIM V® medium (Life Technologies, Tokyo, Japan) was used as serum-free medium for sphere formation assay. Next, the number of spheres was counted, and their diameters were measured in five visual fields (400× magnification) chosen at random.

### Tumor transplantation and measurement of tumor growth

MSTO-211H cells (2 × 10^6^ cells) were incubated with or without 10 *μ*mol/L IMD-0354 overnight and suspended in 200 *μ*L of PBS. Cells incubated with or without IMD-0354 were subcutaneously injected to the right or left side of the shoulders and hips of eight female BALB/c-*nu/nu* mice (purchased from Charles River Japan, Inc., Yokohama, Japan) at the age of 4 weeks. Tumor size was measured twice in a week. The estimated tumor volume was calculated using the following formula: tumor volume = [(width)^2^ × length]/2.

### Mesothelioma orthotopic tumor model

MSTO-211H cells (1 × 10^6^ cells) in 0.2 mL PBS were injected to the thoracic cavity of 15 severe combined immunodeficiency (SCID) mice. One week after inoculation, mice were randomized to one of the following treatment groups: (1) intraperitoneal (i.p.) PBS, daily administration; (2) i.p. IMD-0354, 10 mg/kg daily administration; or (3) i.p. cisplatin, 2 mg/kg daily administration. Mice were sacrificed when they met the established criteria for minimizing pain and suffering. Survival was calculated from the time of inoculation to the date of death. All experiments with animals were performed in compliance with the standards outlined in the guidelines of the University Animal Care and Use Committee of the Tokyo University of Agriculture and Technology.

### Statistical analysis

A two-tailed Student's *t*-test was performed for comparisons of two groups, and one-way analysis of variance or Bonferroni/Dunnett analysis was performed for comparisons of three or more groups. Modified Wilcoxon test was performed for comparison of survival of two groups. *P *<* *0.05 was considered as the level of significance.

## Results

### Constitutive activation of NF-*κ*B in mesothelioma cells was suppressed by treatment with IMD-0354

**Figure 1:**
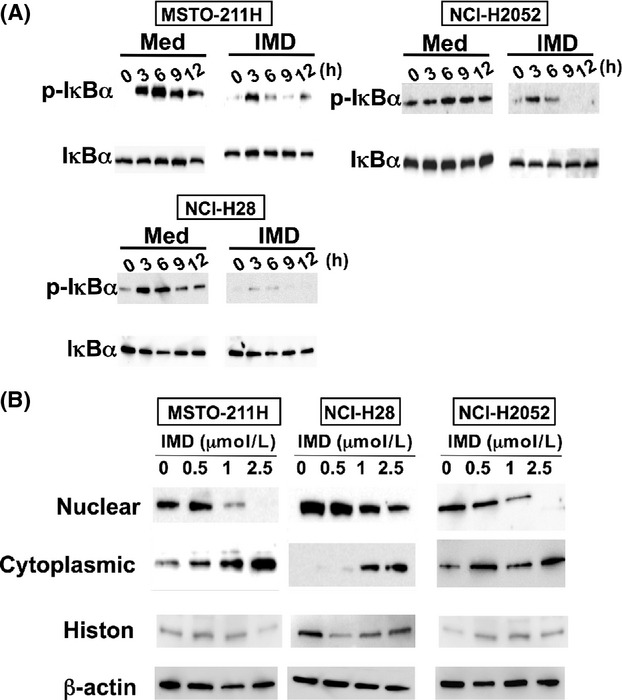
Constitutive activation of NF-*κ*B was suppressed by IKK*β* inhibition via a decrease in I*κ*B*α* phosphorylation. (A) After serum deprivation for 12 h, cells were incubated in flesh medium with 10% fetal bovine serum. At each time point, phosphorylation of I*κ*B*α* was detected with cells treated with diluent alone (Med) or IMD-0354 (IMD, 2 *μ*mol/L). (B) Cells were incubated with each concentration of IMD-0354 (IMD) for 12 h, and the NF-*κ*B p65 subunit was extracted from the nuclear fraction or cytoplasmic fraction. Representative photos in three individual experiments are shown. NF-*κ*B, nuclear factor-*κ*B.

To examine the activity of NF-*κ*B, I*κ*B*α* phosphorylation was assessed in three mesothelioma cell lines: MSTO-211H, NCI-H2052, and NCI-H28. Protein expression was examined by Western blot analysis. I*κ*B*α* phosphorylation was detected in all cell lines, but there were some differences in the expression profile among the cell lines. In MSTO-211H cells, I*κ*B*α* phosphorylation was not detected under the serum-deprived condition, but it was induced 3 h after serum stimulation. Conversely, NCI-H2052 cells exhibited I*κ*B*α* phosphorylation during serum deprivation. Moderate I*κ*B*α* phosphorylation was detected in NCI-H28 cells under the serum-deprived condition. Phosphorylation was apparently decreased when cells were treated with 2 *μ*M IMD-0354 (Fig. 1A). Next, nuclear translocation of the p65 subunit of NF-*κ*B was assessed. The NF-*κ*B p65 subunit was detected in the nuclear fraction of all untreated cells, but in MSTO-211H and NCI-H2052 cells, the p65 subunit was also present in the cytoplasmic fraction. The nuclear fraction of the NF-*κ*B p65 subunit was decreased in a concentration-dependent manner in all cell lines. In particular, 2.5 *μ*mol/L IMD-0354 completely abolished the nuclear fraction of the NF-*κ*B p65 subunit in MSTO-211H and NCI-H2052 cells (Fig. 1B).

### Effect of NF-*κ*B suppression on the proliferation of mesothelioma cells

**Figure 2:**
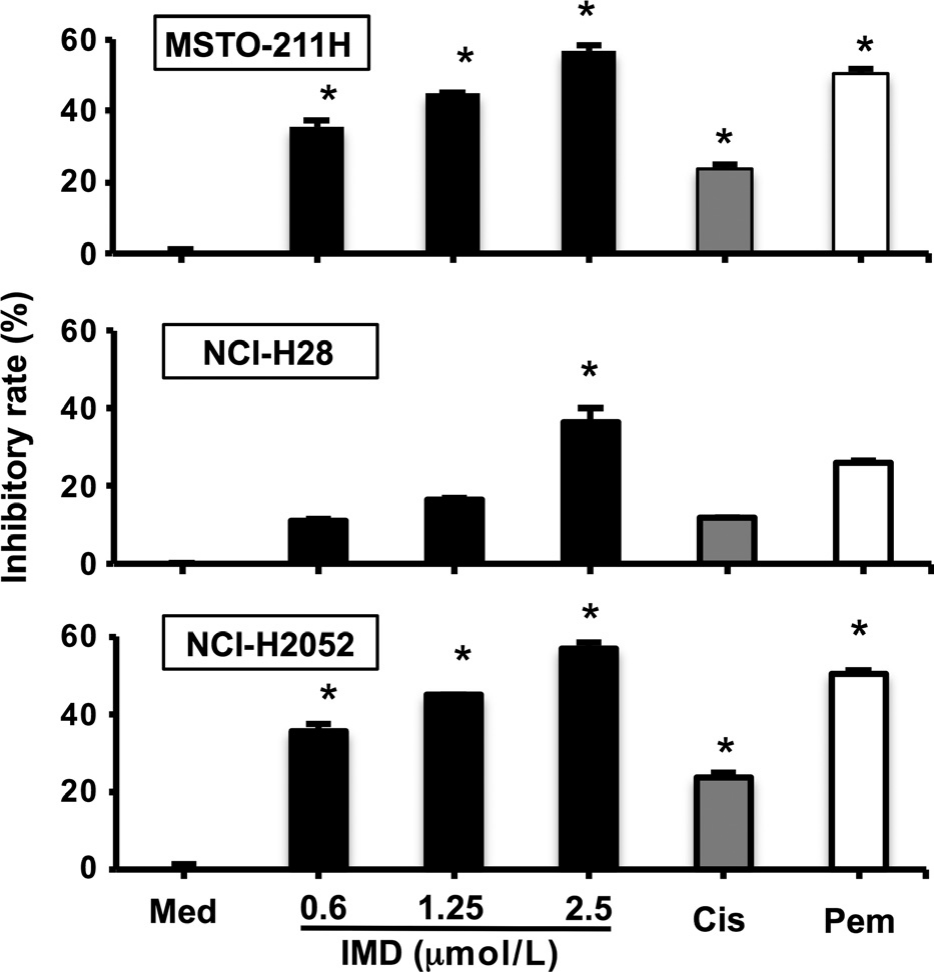
Inhibitory effect of the suppression of NF-*κ*B on the proliferation of mesothelioma cells. MSTO-211H, NCI-H28, and NCI-H2052 cells were incubated in the absense (Med) or the presence of increasing concentrations of IMD-0354 (IMD), cisplatin (Cis, 0.01 *μ*g/mL), and pemetrexed (Pem, 0.1 *μ*g/mL) for 48 h, and the proliferation of cells was determined using the MTT assay. Columns, means of 5–6 different experiments; bars, ±SE. *P < 0.05 compared with medium alone. NF-*κ*B, nuclear factor-*κ*B; MTT, 3-[4,5-dimethylthiazol-2-yl]-2,5-diphenyltetrazolium bromide.

Next, the effect of NF-*κ*B inhibition on the proliferation of mesothelioma cells was evaluated using an MTT assay after 48 h. As shown in Figure 2, the proliferative activities of both MSTO-211H and NCI-H2052 cells were significantly suppressed by treatment with IMD-0354 as well as chemotherapeutic agents (pemetrexed or cisplatin). Significant suppressive effects on the proliferation of NCI-H28 cells were observed at 2.5 *μ*mol/L IMD-0354. However, no additive effects of IMD-0354 with pemetrexed or cisplatin were identified in any of the cell lines (Fig. S1).

### Cell cycle arrest and the downregulation of cell cycle regulatory proteins in mesothelioma cells upon NF-*κ*B inhibition

MTT assay measures cellular activity that reflects proliferation and survival of cells. Therefore, we next checked the effect of NF-*κ*B suppression on cell cycle progression in mesothelioma cells by assessing PI uptake. The cell population at the subG_1_/G_1_ phase in all cell lines treated with 1.25 *μ*mol/L IMD-0354 for 24 h was significantly increased (Table 1). In contrast, cells at the S phase were markedly decreased by IMD-0354 treatment in all cell lines (Table 1). Furthermore, to identify whether IMD-0354 induced apoptosis or not, apoptotic cells were detected by TUNEL assay. Unexpectedly, percentages of TUNEL-positive cells were not increased markedly by treatment with IMD-0354 within 24 h (Fig. S2).

**Table 1:** Distribution of cell cycle after treatment.

Cell line	Treatment	Sub G1/G1 (%)	S (%)	G2/M (%)
MST0-211H	Control	39.8 ± 0.7	58.1 ± 0.1	2.1 ± 0.3
	IMD-0354	54.5 ± 0.5*	42.6 ± 0.3*	2.8 ± 0.1
	Pemetrexed	45.4 ± 0.5*	52.5 ± 0.1	2.1 ± 0.5
NCI-H28	Control	65.1 ± 0.6	31.4 ± 0.3	3.6 ± 0.6
	IMD-0354	69.1 ± 0.4*	27.2 ± 0.5*	3.7 ± 0.4
	Pemetrexed	57.2 ± 0.7	38.3 ± 0.6	4.5 ± 0.1
NCI-H2052 experiments	Control	49.7 ± 0.5	48.9 ± 0.3	2.8 ± 0.1
	IMD-0354	55.4 ± 0.9*	42.8 ± 0.6*	6.3 ± 0.3
	Pemetrexed	48.7 ± 0.3	48.9 ± 0.3	2.4 ± 0.6

Mean ± SE of three individual experiments are shown.

* *P* < 0.05, when compared to control using a Bonferroni/Dunnett comparison.

**Figure 3:**
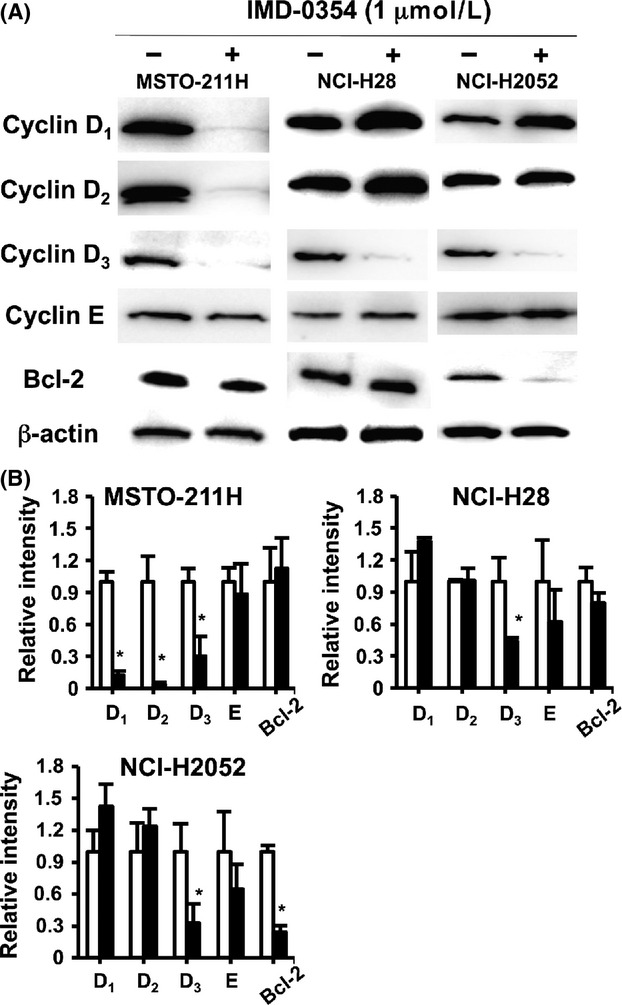
Downregulation of cell cycle regulatory and antiapoptotic proteins in mesothelioma cells upon NF-*κ*B inhibition. (A) The expression of cyclins D1, D2, and D3 was suppressed in MSTO-211H cells and that of cyclin D3 was decreased in NCI-H28 and NCI-H2052 cells by treatment with IMD-0354 (1 *μ*mol/L) for 24 h. In NCI-H2052 cells, Bcl-2 expression was suppressed by IMD-0354 treatment (representative of three different experiments). (B) The relative intensity of each factor was normalized to the protein expression of *β*-actin as an endogenous control. The intensity in the absence of IMD-0354 was scored as 1. D1, cyclin D1; D2, cyclin D2; D3, cyclin D3; E, cyclin E. Columns, means of five independent experiments; bars, ±SE. *P < 0.05 compared with medium alone. NF-*κ*B, nuclear factor-*κ*B.

The expression of cyclins and their contribution to cell proliferation were previously demonstrated in various cell types 21,27. Since NF-*κ*B inhibition resulted in the increase in cell numbers at the subG_1_/G_1_ phase, we examined the expression of cyclins related to the S phase entry in these mesothelioma cell lines. As shown in Figure 3A, cyclins D1, D2, D3, and E were detected in all mesothelioma cell lines, and cyclin D3 expression was significantly decreased by NF-*κ*B suppression in all cell lines. Cyclins D1 and D2 were also significantly downregulated in MSTO-211H cells upon NF-*κ*B inhibition. In contrast, in NCI-H2052 cells, cyclin D1 and D2 expression was increased by NF-*κ*B suppression. Similarly, cyclin D1 was upregulated in NCI-H28 cells upon NF-*κ*B inhibition. At the same time, we assessed the expression of the antiapoptotic protein Bcl-2 in these samples. The expression of Bcl-2 was significantly decreased upon NF-*κ*B inhibition only in NCI-H2052 (Fig. 3).

### Sphere formation was inhibited upon NF-*κ*B suppression

**Figure 4:**
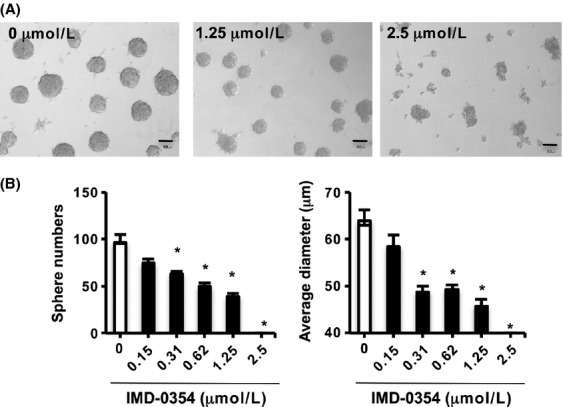
Inhibitiory effect of NF-*κ*B suppression on sphere formation in mesothelioma cells. (A) MSTO-211H cells could form spheres in a serum-deprivation condition. Numbers and diameter of spheres were reduced by treatment of IMD-0354. The bar in the photo indicates 50 *μ*m. Representative photos of 5–6 individual experiments were provided. (B) NF-*κ*B suppression inhibited the growth and survival of sphere-forming cells. Columns, means of 5–6 different experiments; bars, ±SE. *P < 0.05 compared with medium alone. NF-*κ*B, nuclear factor-*κ*B.

Recently, the tumor sphere-culture system has been discussed because of its microenvironmental characteristics that are disadvantageous for the survival and proliferation of cancer cells 28. In addition, patients with mesothelioma often exhibit pleural effusion containing clusters of tumor cells in sphere-like clusters, and these clusters are believed to relate to metastasis to the thoracic cavity in patients with mesothelioma 1. Therefore, we examined the capability of three mesothelioma cell lines to form spheres under serum deprivation. Moreover, we assessed the influence of NF-*κ*B inhibition on sphere formation, especially in MSTO-211H cells, which were most susceptible to NF-*κ*B inhibition. As shown in Figure 4A, only MSTO-211H cells could form spheres and survive under serum deprivation. Therefore, we assessed the influence of NF-*κ*B suppression on sphere formation by measuring the diameter and number of spheres. Sphere formation was suppressed by NF-*κ*B inhibition in an IMD-0354 concentration-dependent manner (Fig. 4A and B).

### Tumor growth was suppressed by NF-*κ*B inhibition

**Figure 5:**
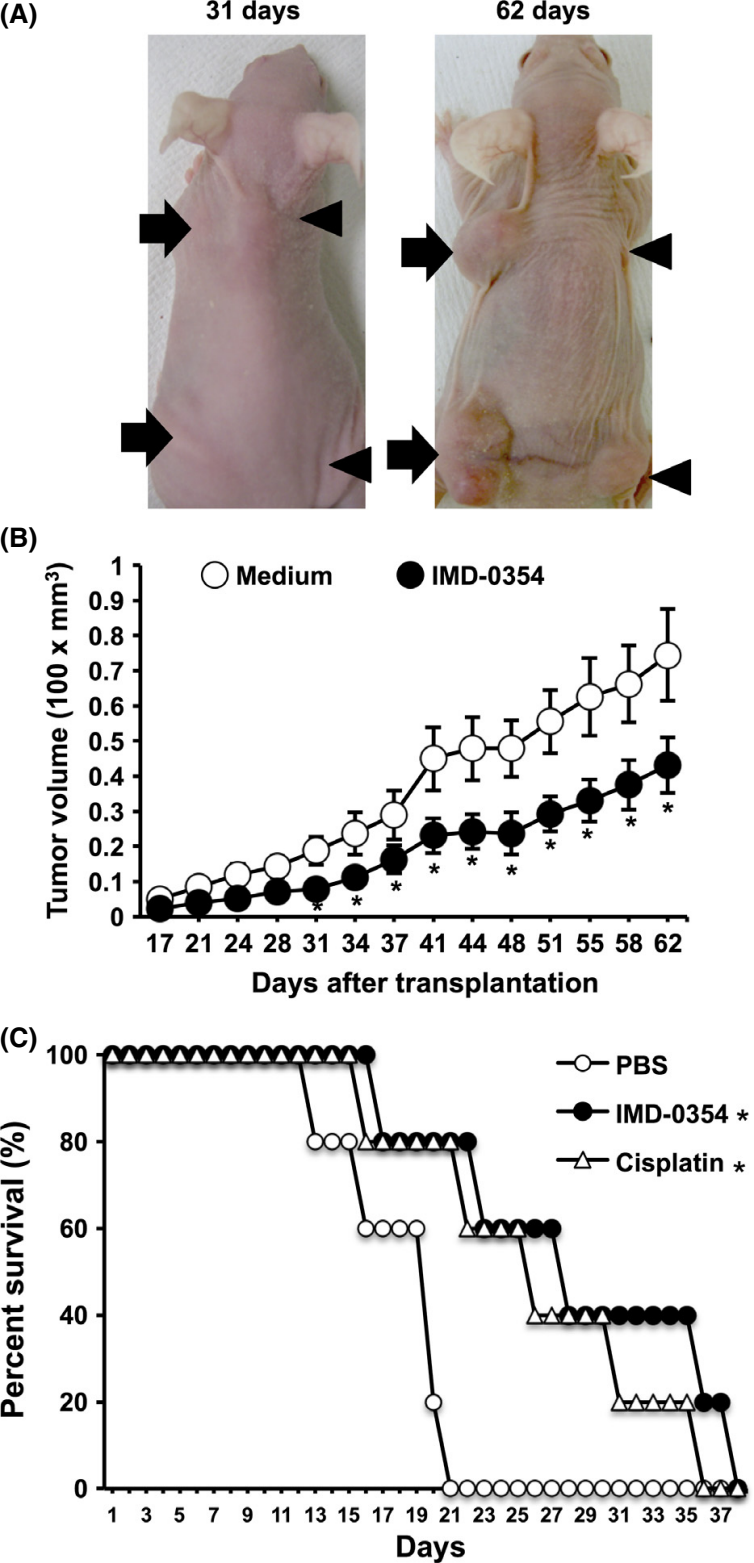
Inhibitory effects of NF-*κ*B inhibition on the in vivo progression of mesothelioma. (A) After incubating MSTO-211H cells with or without IMD-0354 (1 *μ*mol/L) overnight, cells were subcutaneously injected into female BALB/c-*nu/nu* mice as described in the section. Cells incubated with IMD-0354 were injected into two sites in the right side (arrowheads), and cells incubated without IMD-0354 were injected into two sites in the left side (arrow) of mice. Left, 31 days after injection; right, 62 days after injection. (B) The growth of tumors derived from IMD-0354-treated cells was delayed compared to the findings in the absence of treatment. Symbols, means of 16 sites in eight mice; bars, ±SE. *P < 0.05 compared with mice transplanted cells that were incubated without IMD-0354. (C) Therapeutic potency of NF-*κ*B inhibition in an orthotopic model of mesothelioma. MSTO-211H cells were injected into the thoracic cavity of 15 mice. One week after inoculation, mice were treated with PBS (five mice), 10 mg/kg IMD-0354 (five mice), or 2 mg/kg cisplatin (five mice) daily. Survival was calculated from the time of inoculation to death. Modified Wilcoxon test was performed for comparison of survival of each group, and statistical significances were obtained between the IMD-0354-treated group and the PBS-treated group, as well as between the Cisplatin-treated group and the PBS-treated group. No statistical significance was identified between the IMD-0354 treated group and the Cisplatin-treated group. *P < 0.05 compared with mice treated PBS alone. PBS, phosphate-buffered saline; NF-*κ*B, nuclear factor-*κ*B.

Next, we examined the influence of NF-*κ*B inhibition on tumor growth by in vivo experiments using eight BALB/c nude mice. We used MSTO-211H cells because of their susceptibility to NF-*κ*B suppression as shown in our experiments. In the same mice, we observed different rates of tumor growth between the right and left sides (Fig. 5A). The growth of tumors derived from cells treated overnight with 10 *μ*mol/L IMD-0354 was suppressed compared to the findings in the absence of treatment (Fig. 5B). In addition, we injected MSTO-211H cells into the thoracic cavity of SCID mice to examine the effects of IMD-0354 treatment on tumor growth. Mice treated daily with IMD-0354 and cisplatin survived significantly longer than PBS-treated mice (Fig. 5C). On the other hand, no significant difference in survival was observed between the IMD-0354 and cisplatin treatment groups.

## Discussion

In this study, we revealed for the first time that NF-*κ*B was constitutively activated in three human mesothelioma cell lines, and this activation was related to the proliferation and sphere formation of mesothelioma cells and tumor development in vivo. NF-*κ*B is already known to have several important cell functions in various cell types 20–22. However, in mesothelioma cells, the true roles of NF-*κ*B and the underlying factors are not fully understood. The biggest issues faced by patients with mesothelioma are the symptoms of lethal cachexia and difficulty in breathing caused by aggressive tumor progression and the subsequent development of space-occupying lesions in the thoracic cavity 2,3. Therefore, the control of proliferative and metastatic functions is especially important in mesothelioma cells.

We have presented the different profiles of I*κ*B*α* phosphorylation following serum stimulation in each cell line. However, when NF-*κ*B was suppressed by IMD-0354 treatment, cell cycle in these cells was equally suppressed, accompanied by inhibition of the S phase entry. These results suggest that IMD-0354 exhibited suppressive effects on the tumorigenic proliferation of mesothelioma cells, regardless of these different pathologic characters, by blocking cell cycle. NF-*κ*B inhibition rarely involved in strong induction of apoptosis of mesothelioma cells might reveal clinical benefit of the inhibitor in terms of avoidance of serious adverse effects 20.

Previous studies that assessed the relationships between tumorigenic proliferation and cell cycle regulation in mesothelioma cells only focused on cyclin D1 29,31,32. In this study, we showed that NF-*κ*B suppression decreased the expression of cyclins D1, D2, and D3 in MSTO-211H cells and also suppressed the expression of cyclin D3 in NCI-H28 and NCI-H2052 cells. This remarkable downregulation of cyclin D1, D2, and D3 expression in MSTO-211H cells may be associated with the inhibitory effect of NF-*κ*B suppression on cell proliferation and cell cycle progression. Conversely, cyclin D1 and D2 expression in NCI-H2052 cells and cyclin D1 expression in NCI-H28 cells were not suppressed, indicating that a compensatory reaction exists for cyclin D3 suppression. As the cell cycles of both NCI-H2052 and NCI-H28 cells were arrested, NF-*κ*B may regulate cell proliferation and the cell cycle primarily through cyclin D3. The expression of Bcl-2 was decreased by NF-*κ*B suppression in NCI-H2052 cells of 3 cell lines used. NF-*κ*B may also involve in an antiapoptotic reaction, as observed in other cancer cells such as breast cancer cells, in a certain type of mesotheliomas 20.

We performed sphere-culture experiments under serum deprivation as a model for tumor cell clusters in pleural effusions. MSTO-211H cells formed spheres in the serum-deprived condition, and NF-*κ*B suppression inhibited sphere growth. Previous study showed that NF-*κ*B pathway affected cell functions relating metabolic adaptation and cell transformation 32,33. Therefore, NF-*κ*B may have roles in the formation and maintenance of spheres in nutrient-starved circumstances, such as the tumor relating microenvironment of the thoracic cavity. In addition, NF-*κ*B activation may protect mesothelioma cells under disadvantageous conditions and induce the first step of pleural dissemination.

Finally, we evaluated the suppressive effects of IMD-0354 on the in vivo progression of mesothelioma using two xenograft models. In the subcutaneous implanted model, the pretreatment of cells with an NF-*κ*B inhibitor delayed tumor growth. As mesothelioma grows in the thoracic cavity, we injected MSTO-211H cells into the thoracic cavity of SCID mice as an orthotopic model for therapeutic evaluation. IMD-0354 treatment significantly extended the survival of mice compared to PBS injection. In addition, IMD-0354 displayed similar efficacy as cisplatin, a major chemotherapeutic agent for mesothelioma. These in vivo experiments showed that IMD-0354 is a potential therapeutic agent for mesothelioma. According to the result obtained in in vitro experiments, no additive effect on combination of IMD-0354 with cisplatin was shown.

We confirmed that IMD-0354 was not associated with any serious side effects and toxicity in a previous study 20. Direct contribution of NF-*κ*B to tumorigenesis of mesothelial cells has been undefined in clinical cases; however, asbestos-induced chronic inflammation in which NF-*κ*B plays a crucial role has been implicated as the main cause of malignant transformation of mesothelial cells. Therefore, the NF-*κ*B pathway must be an important molecular target in the progression of mesothelioma, and IMD-0354 has a potential role in anti-mesothelioma therapy as well as prevention of mesothelioma formation in patients seriously exposed to asbestos.

## Supporting Information

Figure S1 Inhibitory effect of IMD-0354 combined with other chemotherapeutic reagents on the proliferation of mesothelioma cells. MSTO-211H cells, NCI-H28 cells, and NCI-H2052 cells were incubated with IMD-0354 (IMD, 0.3 μmol/L), cisplatin (Cis, 0.01 μg/mL), and pemetrexed (Pem, 0.1 lg/mL) as indicated for 48 h, and the proliferation of cells were determined by MTT assay. Columns, means of five to six different experiments; bars, mean ± SE.

Figure S2 Effect of IMD-0354 on the apoptosis of mesothelioma cells. MSTO-211H, NCI-H28, and NCI-H2052 cells were incubated in the absence (Med) or presence of IMD-0354 (IMD, 1.25 μmol/L) and pemetrexed (Pem, 0.1 μg/mL) for 24 h. Apoptosis was detected by the TUNEL assay as described in Materials and Methods. Numerals in each photo represents mean ± SE of TUNEL-positive cells in each cell line of five different experiments. *P < 0.05 compared with cells treated diluent alone.

Figure S3 Photos of other three of eight mice subjected to the expariment performed in Figure 5. Cells incubated with IMD-0354 were injected into two sites in the right side (arrowheads), and cells incubated without IMD-0354 were injected into two sites in the left side (arrows) of mice. Upper, 31 days after injection; lower, 62 days after injection.
