# Supplementary figures and images for: A molecular targeting against nuclear factor-κB, as a chemotherapeutic approach for human malignant mesothelioma
Source: Cancer Med. 2014 Feb 10;3(2):416–25. doi: 10.1002/cam4.202 (PMC3987091; doi:10.1002/cam4.202)

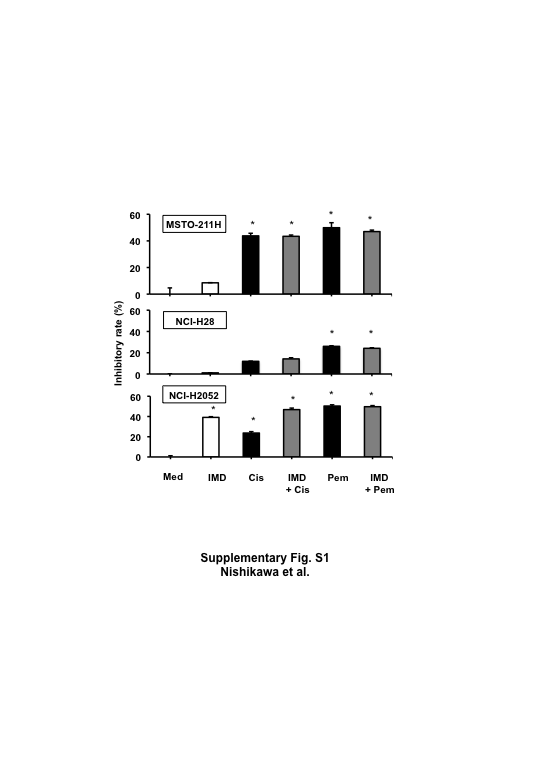

Supplement: Figure S1 — Inhibitory effect of IMD-0354 combined with other chemotherapeutic reagents on the proliferation of mesothelioma cells. MSTO-211H cells, NCI-H28 cells, and NCI-H2052 cells were incubated with IMD-0354 (IMD, 0.3 μmol/L), cisplatin (Cis, 0.01 μg/mL), and pemetrexed (Pem, 0.1 lg/mL) as indicated for 48 h, and the proliferation of cells were determined by MTT assay. Columns, means of five to six different experiments; bars, mean ± SE. [file cam40003-0416-sd1.tif]

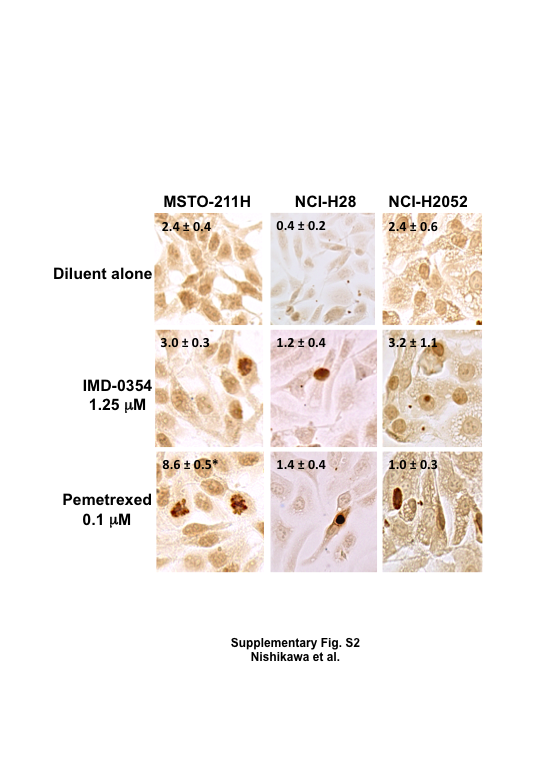

Supplement: Figure S2 — Effect of IMD-0354 on the apoptosis of mesothelioma cells. MSTO-211H, NCI-H28, and NCI-H2052 cells were incubated in the absence (Med) or presence of IMD-0354 (IMD, 1.25 μmol/L) and pemetrexed (Pem, 0.1 μg/mL) for 24 h. Apoptosis was detected by the TUNEL assay as described in Materials and Methods. Numerals in each photo represents mean ± SE of TUNEL-positive cells in each cell line of five different experiments. *P < 0.05 compared with cells treated diluent alone. [file cam40003-0416-sd2.tif]

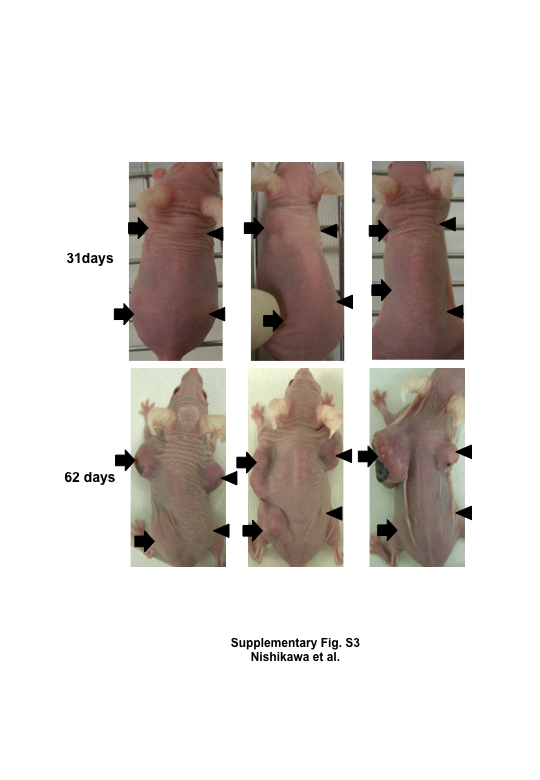

Supplement: Figure S3 — Photos of other three of eight mice subjected to the expariment performed in Figure 5. Cells incubated with IMD-0354 were injected into two sites in the right side (arrowheads), and cells incubated without IMD-0354 were injected into two sites in the left side (arrows) of mice. Upper, 31 days after injection; lower, 62 days after injection. [file cam40003-0416-sd3.tif]
